# Study of complex structural variations of X-linked deafness-2 based on single-molecule sequencing

**DOI:** 10.1042/BSR20203740

**Published:** 2021-06-10

**Authors:** Yi Jiang, Lihua Wu, Shasha Huang, Pidong Li, Bo Gao, Yongyi Yuan, Siwen Zhang, Guoliang Yu, Yong Gao, Hao Wu, Pu Dai

**Affiliations:** 1 Department of Otolaryngology-Head and Neck Surgery, Shanghai Ninth People’s Hospital, Shanghai Jiao Tong University School of Medicine, Shanghai, China; 2 Ear Institute, Shanghai JiaoTong University School of Medicine, Shanghai, China; 3 Shanghai Key Laboratory of Translational Medicine on Ear and Nose Diseases, Shanghai 200092, China; 4 Department of Otolaryngology, Head and Neck Surgery, Institute of Otolaryngology, Chinese PLA General Hospital, Beijing, China; 5 Department of Otolaryngology, Fujian Medical University ShengLi Clinical College, Fujian Provincial Hospital, Fuzhou, China; 6 National Clinical Research Center for Otolaryngologic Diseases, 28 Fuxing Road, Beijing 100853, China; 7 Key Laboratory of Hearing Impairment Science of Ministry of Education, 28 Fuxing Road, Beijing 100853, China; 8 Key Laboratory of Hearing Impairment Prevention and Treatment of Beijing, 28 Fuxing Road Beijing 100853, China; 9 GrandOmics Biosciences, Beijing 100000, China

**Keywords:** genome rearrangements, IP-III, POU3F4, single-molecule sequencing, structural variations, X-linked deafness

## Abstract

X-linked deafness-2 (DFNX2) is cochlear incomplete partition type III (IP-III), one of inner ear malformations characterized by an abnormally wide opening in the bone separating the basal turn of the cochlea from the internal auditory canal, fixation of the stapes and cerebrospinal fluid (CSF) gusher upon stapedectomy or cochleostomy. The causative gene of DFNX2 was *POU3F4*. To investigate the genetic causes of DFNX2 and compare the efficiency of different sequencing methods, 12 unrelated patients were enrolled in the present study. Targeted next-generation sequencing (NGS) and long-read sequencing were used to analyze the genetic etiology of DFNX2. Six variants of *POU3F4* were identified in this cohort by NGS. Three patients with a negative diagnosis based on NGS were enrolled in further long-read sequencing. Two of them were all found to carry structural variations (SVs) on chromosome X, consisting of an 870-kb deletion (DEL) at upstream of *POU3F4* and an 8-Mb inversion (INV). The 870-kb DEL may have been due to non-homologous end joining (NHEJ), while non-allelic homologous recombination (NAHR) within a single chromatid may have accounted for the 8-Mb INV. Common *POU3F4* mutations in DFNX2 included point mutations, small insertions and deletions (INDELs), and exon mutations, which can be detected by Sanger sequencing and NGS. Single-molecule long-read sequencing constitutes an additional and valuable method for accurate detection of pathogenic SVs in IP-III patients with negative NGS results.

## Introduction

X-linked deafness, which accounts for approx. 5% of all cases of congenital deafness, is categorized into seven types according to age of onset and hearing phenotype [1]. To date, five X-linked genes have been found to be related to the etiology of non-syndromic hearing loss (NSHL): *PRPS1* (DFNX1, OMIM:311850), *POU3F4* (DFNX2, OMIM:300039), *SMPX* (DFNX4, OMIM:300226), *AIFM1* (DFNX5, OMIM:300169) and *COL4A6* (DFNX6, OMIM:303631). Among these causative mutant genes for syndromic hearing loss (SHL), *GPRASP2* (DFNX7, OMIM:300969) was shown to be associated with X-linked external auditory canal atresia-dilated internal auditory canal-facial dysmorphism syndrome, while *COL4A5* (OMIM:301050) was implicated in Alport syndrome (OMIM: 301050) and *TIMM8A* (OMIM:300356) in deafness-dystonia-optic neuropathy syndrome (Mohr–Tranebjærg syndrome OMIM: 304700).

X-linked deafness-2 (DFNX2) was first reported as X-linked deafness with stapes fixation (DFN3) [2]. Patients with DFNX2 exhibit inner ear malformation, characterized by an abnormally wide opening in the bone separating the basal turn of the cochlea and the internal auditory canal, fixation of the stapes, and cerebrospinal fluid (CSF) gusher upon stapedectomy or cochleaostomy [3]. Further familial evidence suggested that the deafness phenotypes caused by variant of the *POU3F4* gene, characterized by progressive conductive and sensorineural hearing loss. High-resolution computed tomography (CT) showed that the basal turn of the cochlea was incompletely separated from the dilated internal auditory canal (internal auditory meatus; IAM), along with an absence of the modiolus and interscalar septum; this condition was designated as cochlear incomplete partition type III (IP-III) [4].

*POU3F4* (GRCh37.p13 ChrX (NC_000023.10): 83508261–83512127) on chromosome Xq21 encodes a transcription factor that binds DNA through two specific subdomains: a POU-specific domain and a POU Homeobox domain. Mutations within the exons of the gene have been described and are believed to disrupt DNA binding ability [5]. Sixty-two mutations in *POU3F4* have been reported since the initial report [6]. In addition, deletions (DEL), inversions (INV), and duplications (DUP) upstream of the *POU3F4* gene were reported to cause DFNX2. The complex structural variations (SVs) were analyzed by fluorescence *in situ* hybridization (FISH), pulsed field gel electrophoresis (PFGE), the multiplex ligation-dependent probe amplification (MLPA) method, and Southern blotting, but the sequences of the breakpoints were difficult to analyze due to the low resolution of SVs detection [7–12].

Here, we present gene data from subjects with CT findings characteristic of IP-III. Targeted next-generation sequencing (NGS) can aid in detection of point mutations and small insertions and deletions (INDELs), while single-molecule sequencing can determine complex SVs more easily and precisely than NGS.

## Materials and methods

### Patients and clinical evaluation

From 2016 to 2018, 12 unrelated male patients with IP-III were enrolled in the present study. All the patients had characteristics of IP-III, i.e., the basal turn of the cochlea was incompletely separated from the dilated IAM, along with an absence of the modiolus and interscalar septum. This was diagnosed by temporal bone CT (axial view) as previous study [13]. The patients were aged from 5 months to 11 years (average age: 3.6 years). A complete physical examination, including audiometry [13], was performed in all patients.

### Ethics statement and DNA samples

This investigation was performed with the approval of the Ethics Committees of the Chinese PLA General Hospital l (approval number: S2016-103-01, date: 2016-09-03 [V1.0]). The parents/guardians of all subjects provided written informed consent prior to blood sampling. DNA was extracted from peripheral blood leukocytes using a commercially available DNA extraction kit (Watson Biotechnologies Inc., Shanghai, China). The families who volunteered to undergo the single-molecule sequencing analysis returned for blood sampling, and genomic DNA was extracted from peripheral blood leukocytes using QIAamp DNA Mini Kit (Qiagen, Valencia, CA, U.S.A.).

### Methods

#### Targeted NGS

An NGS panel of 109 human deafness genes, including all reported genes for NSHL and some relatively common genes for SHL (at the time when the panel was designed) (Supplementary Table S1) were selected [14]. This panel was designed using biotinylated oligonucleotide probes to capture all exons of 109 genes, and their splice sites with the flanking 50-bp intron sequences. Captured DNA fragments were sequenced (HiSeq2000; Illumina, San Diego, CA, U.S.A.). Data analysis and bioinformatics processing were performed according to the standard Illumina procedures. Reads were aligned to the NCBI37/hg19 assembly using the BWA Multi-Vision software package (http://sourceforge.net/projects/bio-bwa/). Single-nucleotide variations (SNVs) and INDELs were detected and genotyped with the GATK Haplotype Caller. Potentially pathogenic variants were classified as nonsense, missense, splice-site, or INDELs variants with allele frequencies < 0.01, as determined by searches of databases including NCBI dbSNP (http://www.ncbi.nlm.nih.gov/projects/SNP), 1000 Genomes [15] and the guidelines of the American College of Medical Genetics and Genomics (ACMG). Sanger sequencing was used to verify the variance in all patients and mothers according to a standard protocol (BigDye Terminator v3.1 Cycle Sequencing Kit, Applied Biosystems by Life Technologies) [16]. The primers used for amplification and sequencing were listed in Supplementary Table S2.

#### Nanopore long-read single-molecule sequencing

Large-insert long-read sequencing libraries were prepared according to the instructions provided by Oxford Nanopore Technologies (ONT, Oxford, U.K.). Briefly, genomic DNA fragments were obtained via g-TUBE (#520079, Covaris, Boston, MA, U.S.A.) and Blue Pippin (Sage Science, Beverly, MA, U.S.A.), and the Ligation sequencing 1D kit (SQK-LSK109, ONT). Nanopore long-read sequencing was performed on PromethION sequencers with R9.4 flow cells (#FLO-MIN106; ONT). Raw data were collected as fast5 files, and base calling was performed using guppy ONT 2.0.8(ONT). Base-called data that passed quality control (quality score ≥ 7) were aligned to the hg19 human reference genome using NGMLR 0.2.7 (https://github.com/philres/ngmlr). SVs were named using Sniffles (https://github.com/fritzsedlazeck/Sniffles). The major types of SVs included DEL, INS, DUP, INV, and translocation (TRA) variants. The ANNOVAR (version 24 October 2019) (http://annovar.openbioinformatics.org/en/latest/user-guide/download/) was applied to annotate the breakpoints of SVs, including genes and functional regions using the database of refGene (http://www.openbioinformatics.org/annovar/download/hg19_refGene.txt.gz). The criteria for annotation of the public SVs/CNVs databases (1000 genome phase3 (ftp://ftptrace.ncbi.nih.gov/1000genomes/ftp/phase3/integrated_sv_map/ALL.wgs.integrated_sv_map_v2.20130502.svs.genotypes.vcf.gz), the Database of Genomic Variant gold standard CNV (http://dgv.tcag.ca/dgv/docs/DGV.GS.March2016.50percent.GainLossSep.Final.hg19.gff3), dbVar:nstd37 (ftp://ftp.ncbi.nlm.nih.gov/pub/dbVar/data/Homo_sapiens/by_study/vcf/nstd37.GRCh37.variant_call.vcf.gz) and Decipher (https://decipher.sanger.ac.uk/files/downloads/population_cnv.txt.gz) were chosen to follow. The two SVs from the same type including deletions, duplication, and inversion were considered the same if they had at least 50% reciprocal overlap (the overlapped region was more than 50% of both calls). Two insertions or translocations were considered the same if the two breakpoints were within 1000 bp.

#### Polymerase chain reaction and Sanger sequencing

We performed polymerase chain reaction (PCR) to detect the presence or absence of a genomic deletion in the genomics of family J0012 or family J0007. Genomic DNA was extracted from blood using a DNA extraction kit (Tiangen Biotech), and PCR amplification was performed as previous study [16]. There were four pairs of primers used for amplification of 870-kb DEL in J0012 family, F4/R4 and F5/R5 were designed to be located on both sides of the junction site of the DEL to detect the mutated DNA fragments; while f1/r1 and f2/r2 were designed to be located on upstream and downstream of the breakpoint of DEL, respectively. Primers sequences were shown in Supplementary Table S4. The primer sequence used to verify the 6-kb DEL is shown: chrX-F: 5′-GTG AGT AGC AGG TGC TTA AT-3′, chrX-R: 5′-CAG TGG CAG TCC ATT TCA TA-3′. The electrophoretic analysis was performed to visualize the PCR results. The PCR products were confirmed using Sanger sequencing.

## Results

### Results of NGS

Six variants of *POU3F4* were identified in this cohort, five of which were novel variants and were not present in dbSNP or 1000 Genomes. The coverage of targeted regions was 98.3%, and the average depth was 281.3-fold. These variants were verified by Sanger sequencing (Supplementary Figure S1), and the variants were all located upstream of the POU-specific domain. According to the guidelines of ACMG, two truncated mutations (p.Q78* and p.V141*) and four frameshift DELs (p.Q136Lfs*58, p.S117Rfs*26, p.H147Qfs*94, and p.A116Gfs*77) were considered pathogenic (Table 1).

**Table 1:** NGS results from our center

No.	Age	Gene	Nucleotide change	Amino acid change	Novel	ACMG criteria	Feature of deafness	Relationship
4238	1 year		NA				SNHL	
4354	1 year	POU3F4	c.346_350dup	p.S117Rfs*26	Yes	PVS1_Strong, PM2(LP)	SNHL	MATERNAL
5517	3 years	POU3F4	c.421_422delinsTA	p.V141*	Yes	PVS1_Strong, PM2(LP)	Mixed	MATERNAL
12701	1 year	POU3F4	c.441del	p.H147Qfs*94	Yes	PVS1_Strong, PM2(LP)	SNHL	NA
13276	2 years	POU3F4	c.346dup	p.A116Gfs*77	Yes	PVS1_Strong, PM2(LP)	NA	NA
14548	8 years		NA				SNHL	
M32	5 months	POU3F4	c.232C>T	p.Q78*	No (Clinvar: 426228)	PVS1_Strong, PM2(LP)	NA	MATERNAL
M40	7 years		NA				NA	
J0006	6 months	POU3F4	c.401_404dup	p.Q136Lfs*58	Yes	PVS1_Strong, PM2(LP)	SNHL	MATERNAL
J0007	11 years		NA				SNHL	
J0011	2 years		NA				SNHL	
J0012	6 years		NA				SNHL	

Abbreviation: SNHL, sensorineural hearing loss. NA in nucleotide change means no available NGS test; NA in feature of deafness means no audiogram.

### Results of nanopore long-read single-molecule sequencing

NGS was negative in the probands in three families (J0007, J0011, and J0012), and the patients’ guardians agreed to undergo single-molecule sequencing testing. The guardians of three other patients with negative NGS results refused the further testing because of the requirement to redraw blood for long DNA fragment extraction. We obtained a total of 40–50 Gb of reads in each sample with a mean length of 11.6–22 Kb, average coverage of 96.7%, and average depth of 10–15× for the whole genome. Among all samples, the mean number of pass reads was 3289010. Typically, >85% of reads were mapped to the reference genome (Supplementary Table S3) [17].

**Figure 1:**
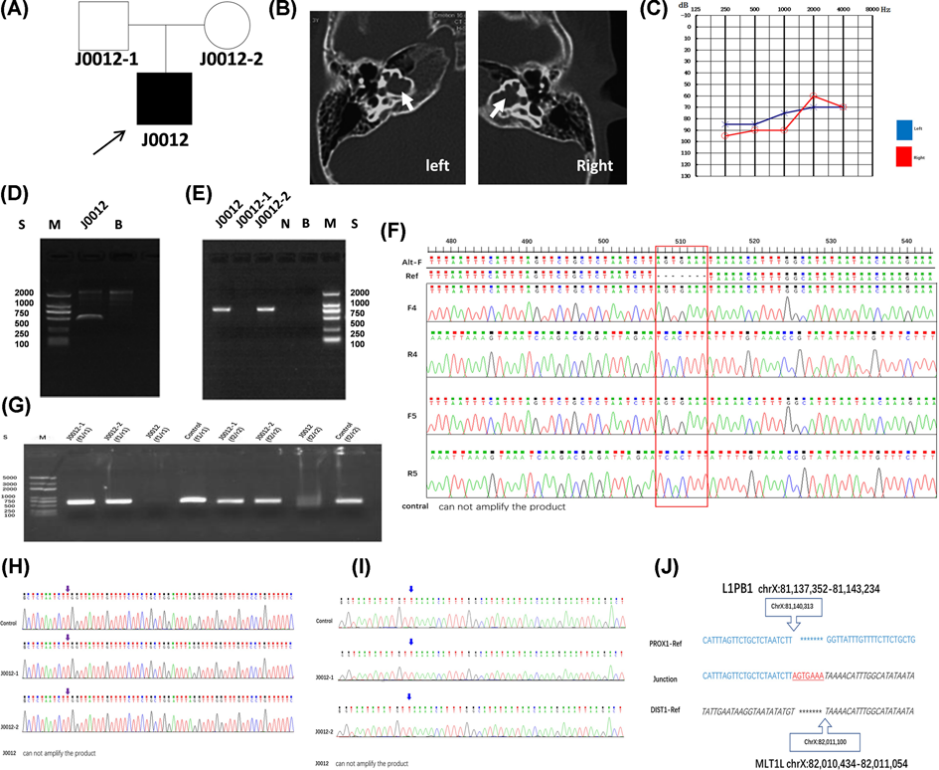
Genetic analysis of J0012 (**A**) Pedigrees of the J0012 family. (**B**) CT of the proband. The white arrow indicates the site of incomplete partition. (**C**) Pure tone audiogram. (**D**) Electrophoresis analysis of a 515-bp product in proband J0012 after amplification with primers F5/R5. (**E**) Electrophoresis analysis of an 812-bp product in the J0012 family. Sample from three members of the J0012 family after amplification with primers F4/R4. (**F**) Sanger sequencing of junction revealed seven novel added nucleotides (AGTGAAA) in the breakpoint region after amplification with primers F4/R4 and F5/R5. (**G**) Electrophoresis analysis of the J0012 family. Samples from the J0012 family and the control were amplified with primers f1/r1 and f2/r2. (**H**) Sanger sequencing of the upstream breakpoint primers (f1/r1). (**I**) Sanger sequencing of the downstream breakpoint primers (f2/r2). (**J**) Alignment of the sequenced junctions in J0012 with the reference genome sequence. Proximal and distal reference sequences are shown in normal font and italics, respectively. The junction is underlined and shown in red. Proximal (top, blue) and distal (bottom, black) sequences were aligned against the junction sequence (middle), including seven novel nucleotides (AGTGAAA), called the‘information scar’, at the junction between the distal and proximal sequences, which is characteristic of NHEJ. (M: Marker; N: normal control (HX1); B: Blank; S: Band Size. Alt-F: alteration sequence with primers of F; Ref: reference sequence; F: forward primer; R: reverse primer; control: normal control group.)

In family J0012, the proband was a 6-month-old male with congenital profound sensorineural hearing impairment (Figure 1A). This proband demonstrated an abnormal dilatation of IAM as well as an abnormally wide communication between the IAM and the inner ear compartment, and exhibited typical audiometric features of mixed hearing impairment (Figure 1B,C). Using Nanopore sequencing, we detected an 870-kb DEL located at ChrX:81140313–82011100, approximately 750 kb upstream of the *POU3F4* gene. A total of seven reads showed the DEL, and the read length was between 7.7 and 18.2 kb. The junction sequence was successfully amplified and sequenced in the proband and his mother (Figure 1D,E). The sequencing data revealed seven novel nucleotides (AGTGAAA) added at the join points (Figure 1F). The PCR results for the upstream and downstream breakpoints in ChrX:81140313–82011100 of J0012 family and controls also verified this DEL (Figure 1G). No significant homology was found within 2 kb on either side of the junction but an L1PB1 element was located at ChrX:81137352–81143234 of the proximal breakpoint with an MLT1L element located at ChrX:82010434–82011054 of the distal breakpoint in the reference human genome sequence (as determined by BLAST2 analysis; Figure 1H–J).

**Figure 2:**
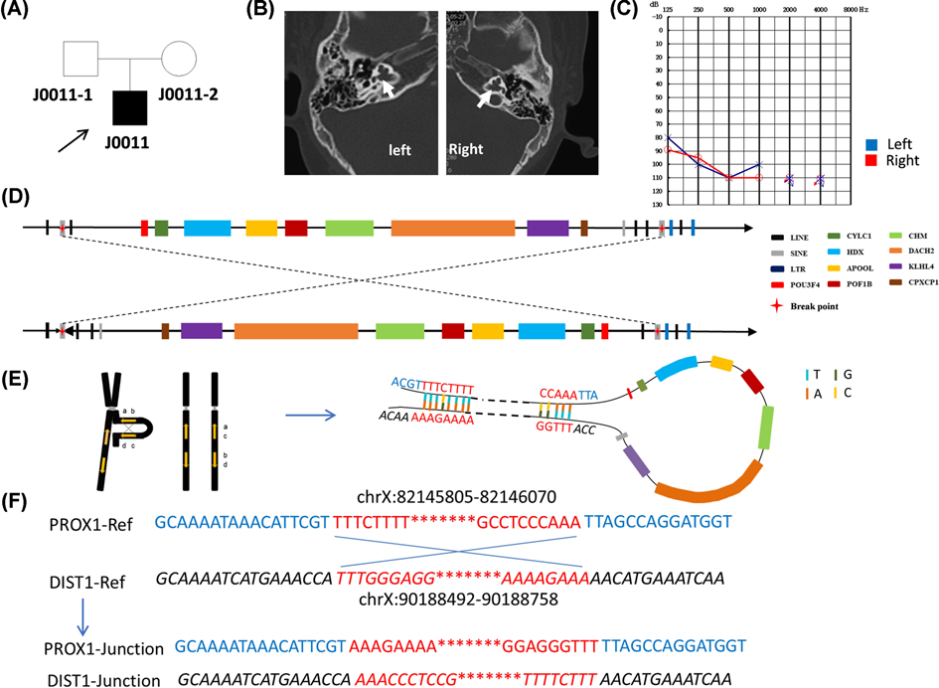
Genetic analysis of J0011 (**A**) Pedigrees of the J0011 family. (**B**) CT scan of the proband. (**C**) Behavioral audiometry. (**D**) The inversion of family J0011. Arrowheads indicate the direction of DNA relative to the positive strand. The genes in this region are shown in different colors. (**E**) RCM sequences were found in the flanking intergenic regions of ChrX:82145805–82146070 and ChrX:90188492–90188758. (**F**) Alignment of the sequenced junctions in J0011 with the reference genome sequence. Proximal and distal reference sequences are shown in normal font and italics, respectively, and in different colors. The RCM sequences are shown in red.

In family J0011, the proband was a 2-year-old male (Figure 2A). This proband demonstrated congenital profound sensorineural hearing impairment with a basal turn of the cochlea was incompletely separated from the dilated (Figure 2B), and the bone conduction and air conduction thresholds were 60 dB in both ears (Figure 2C). We found an 8-Mb INV located at ChrX:82146071–90188492, which was supported by a total of seven reads. The INV included *POU3F4, CYLC1, HDX, APOOL, POF1B, CHM, DACH2, KLHL4*, and *CPXCR1* genes (Figure 2D). While this INV was not detected by karyotype analysis (data not shown). The proband only showed IP-III, with no changes in visual acuity or dysgnosia. Sequence analysis of 2-kb distal and proximal breakpoints revealed Alu elements with a core sequence of GCTGG, highly dense regions of retrotransposons (LTRs, LINE-1, SINE), a Chi sequence (GCTGGTGG) located at the proximal breakpoint 457 bp from the junction, and reverse complementary match (RCM) sequences at ChrX:82145805–82146070 and ChrX:90188492–90188758 (Figure 2E,F).

**Figure 3:**
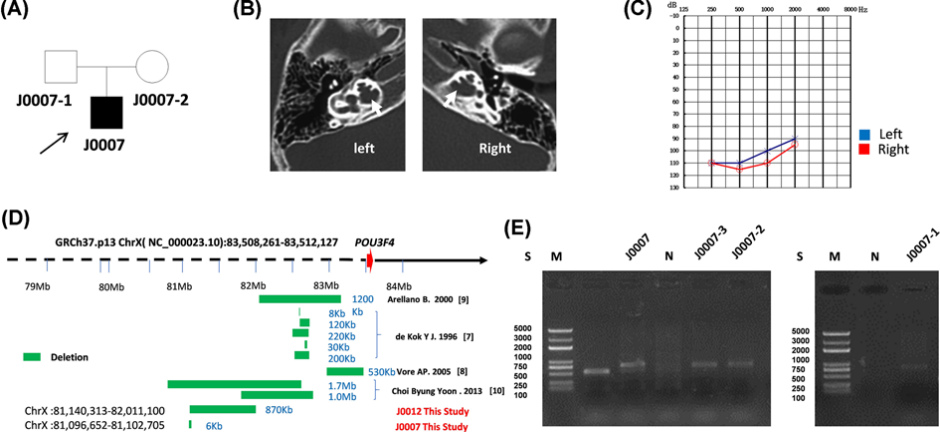
Genetic analysis of J0007 (**A**) Pedigrees of the J0007 family. (**B**) CT scan of the proband. The white arrow indicates the site of incomplete partition. (**C**) Pure-tone audiogram. (**D**) The deletion within the flanking region of *POU3F4* in families J0012 and J0007, and in previous reports. (**E**) Electrophoresis analysis of the J0012 family.

In family J0007, the proband was an 11-year-old boy (Figure 3A). This proband demonstrated a dilatation of the lateral end of the IAM and a deficit or absence in the basal turn of the cochlea (Figure 3B), and exhibited congenital profound sensorineural hearing impairment (Figure 3C). Using Nanopore sequencing, we detected a 6-kb DEL located at ChrX:81096652–81102705 at a region with a high density of retrotransposons and an L1Hs element (LINE family), supported by a total of five reads (Figure 3D). While PCR combined with Sanger sequencing failed to verify this 6-kb DEL (Figure 3E, sequence data not shown), indicating that this 6-kb DEL may be a negative result.

## Discussion

NGS of a targeted gene panel and whole-exome sequencing are now widely used for genetic testing of patients with suspected or clinically proven genetic disorders [18]. Using a panel of 109 reported human deafness genes, we found that 6 of 12 patients with IP-III carried mutations in the *POU3F4* coding region. Three patients with a negative diagnosis following NGS were then examined by single-molecule sequencing. There was an 870-kb DEL of chromosome X at upstream of *POU3F4* and an 8-Mb INV including *POU3F4*. Single-molecule long-read sequencing is valuable for accurate detection of pathogenic SVs in patients with negative NGS results.

Negative NGS findings may occur due to short-read sequencing and GC-bias, inaccurate interpretation of genetic variants, the inability to span long repetitive elements, the low sensitivity of SV detection, and the high false-positive rate [19]. Among these reasons, repetitive elements that are widespread in the genome, such as low copy repeats (LCRs), segmental duplications (SDs), short interspersed nuclear elements (SINEs), and long interspersed nuclear elements (LINEs), may play important roles in SVs formation [20]. To detect the underlying cause of SVs, MLPA, FISH, gene chip, and CNV-seq are commonly used [7–11]. However, these techniques cannot reveal consecutive sequences up to 1-kb within a single allele.

Sequence analysis can help us to understand genome rearrangements including SV formation. Genome rearrangements caused by genomic structural instability and SV formation can be categorized into two major groups: recurrent or non-recurrent rearrangements [21]. Recurrent rearrangements occur in multiple unrelated individuals and are caused by non-allelic homologous recombination (NAHR) between relatively large (>10 kb) DNA repeats, such as LCRs or SDs, and share ≥97% sequence identity. Non-recurrent rearrangements occur at a single locus and can be caused by several mechanisms, including non-homologous end joining (NHEJ), retrotransposition or NAHR between repetitive sequences, such as L1 and Alu elements [22].

DUP/INV events have been reported by de Kok et al. [12] using FISH, PFGE, and Southern blotting analyses. This study revealed a complex rearrangement in the Xq21.1 region, and presumably also in the Xq21.3 region. Using single-molecule sequencing, we examined the genomic positions where the proposed template switching occurred in J0011 and superimposed these locations on the regional genome architecture. Intrachromatid recombination events between LCRs can lead to an INV when the LCRs are in an inverted orientation and NAHR occurs within a single chromatid. NAHR between paralogous sequence repeats, such as RCMs, is the predominant mechanism underlying recurrent, and some non-recurrent, genomic rearrangements; other mechanisms involving variable clustered breakpoints, which were thought to repair double-strand DNA breaks (DSBs) in late S or G_2_ of the cell cycle, have also been implicated [23,24] (Figure 2). Human *POU3F4* gene is located in a 3-Mb gene desert region enriched in highly conserved non-coding regions (HCNRs). HCNRs enriched in *cis*-regulatory elements are related to strict regulation of developmental gene expression patterns [25]. It has been determined that the 920-kb region upstream of the *POU3F4* gene contains cis-regulatory sequences that are essential for the expression of *POU3F4* during the development of the inner ear [26]. The 8-Mb INV started approx. 1 Mb upstream of the *POU3F4* gene. Studies have reported that several enhancers of HCNRs at the 1 Mb upstream of the *POU3F4* gene affects the expression of *POU3F4* and participates in the inner ear development of *Xenopus* and zebrafish [26,27]. Therefore, we speculate that this 8-Mb INV may destroy the regulatory region, thereby affecting the function of *POU3F4* and inner ear development.

NHEJ is the primary pathway for the repair of DSBs caused by ionizing radiation or oxidative free radicals, in human cells and in multicellular eukaryotes throughout the cell cycle. NHEJ is an imperfect process because several nucleotides at each end of the DNA break are lost in most instances [28]. In family J0012, sequence analysis of the breakpoint revealed non-homologous breakpoint regions with no repetitive elements, but an L1PB1 element of the proximal breakpoint with an MLT1L element of the distal breakpoint. With the exception of the 870-kb DEL, the sequencing data identified a 7-bp sequence (AGTGAAA) as the ‘information scar’ at the joining points, indicating NHEJ as a DEL mechanism. (Figure 1) There is an enhancer-enriched region 1 Mb upstream of the *POU3F4* gene. Deafness phenotypes were associated with upstream chromosomal rearrangements, including transcriptional silencing, due to a positional effect (separation of *POU3F4* from an upstream enhancer element) [29,30]. This may have been detected by NGS if a suitable capture probe for this area had been available. Individuals with DFNX2 and abnormalities upstream of *POU3F4* have been reported previously [31].

Deafness caused by the *POU3F4* mutation accounts for nearly 50% of X-linked nonsyndromic deafness [16]. A variety of pathogenic mutations within and upstream of the *POU3F4* gene have been identified [16]. c.499C>T leads to premature termination of *POU3F4* translation before the entire POU domain [32]. INV(X)(q21.1q22.3) promotes the centromere localization in *POU3F4* gene, causing DFNX2 [31]. The probands included in this study all had typical symptoms of non-syndromic deafness, and the source of gene mutations has not been fully confirmed. In Asian populations, in addition to inheritance, IP-III cases can also occur through *de novo* mutations at the DFNX2 locus. Compared with point mutations at the DFNX2 locus, large deletions in the genome are more likely to occur in *de novo* [33]. Therefore, in follow-up studies, we not only need to determine the source of the variation, but also need to confirm the relationship between the variation and the disease onset.

## Conclusion

Mutations involving the *POU3F4* gene have been found to cause IP-III, an inner ear malformation with distinctive characteristics including an abnormally wide opening in the bone separating the basal turn of the cochlea from the internal auditory canal. The most common variants are point mutations, small INDELs or exons mutations that can be detected by Sanger sequencing and NGS. Single-molecule long-read sequencing is valuable for accurate detection of pathogenic SVs in patients with negative NGS results. Using this technology, the accuracy of IP-III diagnosis has greatly improved. Moreover, sequences around the breakpoint can be explored, which will be useful for determination of the mechanisms underlying SV formation.

## Supplementary Material

Supplementary Figure S1 and Tables S1-S4

## Data Availability

The data used to support the findings of the present study were supplied by the author P.D. under license and so cannot be made freely available. Requests for access to these data should be made to P.D. (daipu301@vip.sina.com).

## Abbreviations

ACMG: American College of Medical Genetics and Genomics
CT: computed tomography
DEL: deletion
DFNX2: X-linked deafness-2
DSB: double-strand DNA break
DUP: duplication
FISH: fluorescence *in situ* hybridization
HCNR: highly conserved non-coding region
IAM: internal auditory meatus
INDEL: insertions and deletion
INV: inversion
IP-III: incomplete partition type III
LCR: low copy repeat
MLPA: multiplex ligation-dependent probe amplification
NAHR: non-allelic homologous recombination
NGS: next-generation sequencing
NHEJ: non-homologous end joining
NSHL: non-syndromic hearing loss
PCR: polymerase chain reaction
PFGE: pulsed field gel electrophoresis
RCM: reverse complementary match
SD: segmental duplication
SHL: syndromic hearing loss
SV: structural variation
