# Supplementary material for: Study of complex structural variations of X-linked deafness-2 based on single-molecule sequencing
Source: Biosci Rep. 2021 Jun 10;41(6):BSR20203740. doi: 10.1042/BSR20203740 (PMC8193640; doi:10.1042/BSR20203740)
Supplement: Supplementary Figure S1 and Tables S1-S4 [file BSR-2020-3740_supp.pdf]

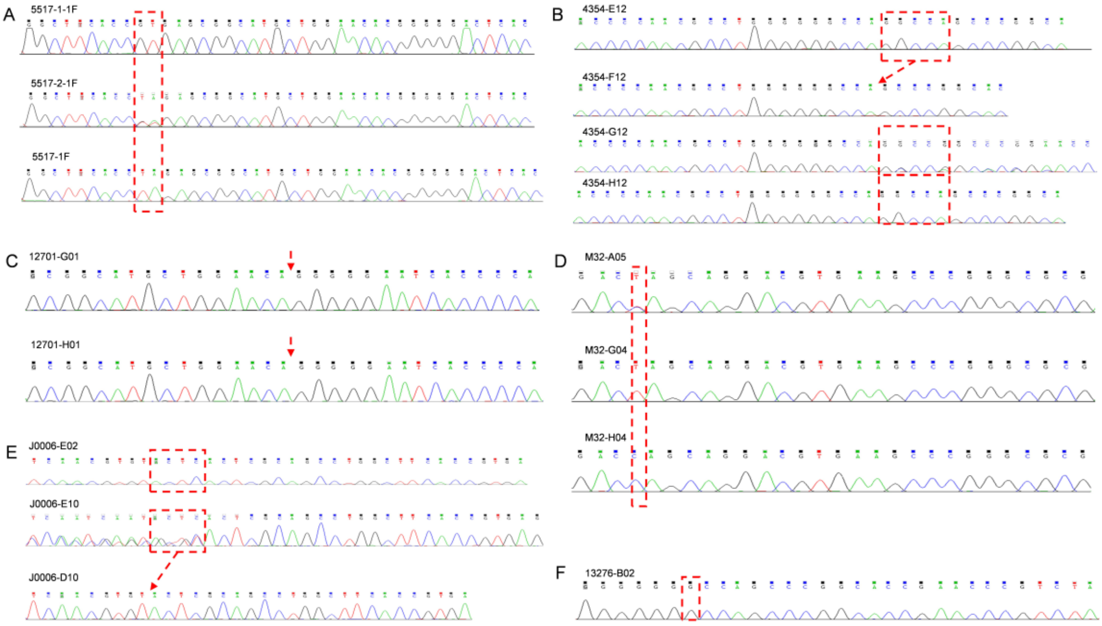
A
5517-1-1F
5517-2-1F
5517-1F
B
4354-E12
4354-F12
4354-G12
4354-H12
C
12701-G01
12701-H01
D
M32-A05
M32-G04
M32-H04
E
J0006-E02
J0006-E10
J0006-D10
F
13276-B02

Supplementary Figure 1: Sanger sequencing results of Six variants in exon of *POU3F4* gene.

A: The hemizygotic c.421_422delinsTA (p.V141*) mutation was identified in the proband (5517-1F), the mother (5517-2-1F) was heterozygous for c.421_422delinsTA (p.V141*) mutation, the father (5517-1-1F) carried the wild-type sequence of *POU3F4* including position 421_422.

B: The c.346_350dup (p.S117Rfs*26) mutation was identified in the proband (4354-E12), the mother (4354-G12), and the grandfather (4354-H12); but not identified in the father (4354-F12).

C: The c.441del (p.H147Qfs*94) mutation was identified in the proband (12701-G01) and the mother (12701-H01); Arrows indicate the deletion site.

D: The c.232C>T (p.Q78*) mutation was identified in the proband (M32-A05) and the mother (M32-G04), the father (M32-H04) carried the wild-type sequence of *POU3F4* including position 232.

E: The c.401_404dup (p.Q136Lfs*58) mutation was identified in the proband (J0006-E02) and the mother (J0006-E10), but not identified in the father (J0006-D10).

F: The c.346dup (p.A116Gfs*77) mutation was identified in the proband (13276-B02).

Supplementary Table 1：The 109 deafness genes

Deafness Genes							
ACTG1	*BSND*	*CABP2*	*CCDC50*	*CDH23*	*CEACAM16*	*CHD7*	*CIB2*
CLDN14	*CLPP*	*CLRN1*	*COCH*	*COL11A1*	*COL11A2*	*COL2A1*	*COL4A3*
COL4A4	*COL4A5*	*COL4A6*	*COL9A1*	*COL9A2*	*CRYM*	*DFNA5*	*DFNB31*
DFNB59	*DIABLO*	*DIAPH1*	*DIAPH3*	*DSPP*	*EDN3*	*EDNRB*	*ELMOD3*
ESPN	*ESRRB*	*EYA1*	*EYA4*	*FOXI1*	*GIPC3*	*GJB2*	*GJB3*
GJB6	*GPR98*	*GPSM2*	*GRHL2*	*GRXCR1*	*HARS2*	*HGF*	*HSD17B4*
ILDR1	*KARS*	*KCNE1*	*KCNJ10*	*KCNQ1*	*KCNQ4*	*LARS2*	*LHFPL5*
LOXHD1	*LRTOMT*	*MARVELD2*	*MITF*	*MSRB3*	*MYH14*	*MYH9*	*MYO15A*
MYO1A	*MYO3A*	*MYO6*	*MYO7A*	*NDP*	*OTOA*	*OTOF*	*P2RX2*
PAX3	*PCDH15*	*PDZD7*	*PNPT1*	*POLR1C*	*POLR1D*	*POU3F4*	*POU4F3*
PRPS1	*PTPRQ*	*RDX*	*SEMA3E*	*SERPINB6*	*SIX1*	*SIX5*	*SLC17A8*
SLC26A4	*SLC26A5*	*SMPX*	*SNAI2*	*SOX10*	*STRC*	*TBC1D24*	*TCOF1*
TECTA	*TJP2*	*TMC1*	*TMIE*	*TMPRSS3*	*TNC*	*TPRN*	*TRIOBP*
TSPEAR	*USH1C*	*USH1G*	*USH2A*	*WFS1*			

Supplementary Table 2：Primers used for amplification and sequencing of *POU3F4*

Patient	Nucleotide change	Forward primer sequence (5'-3')	Reverse primer sequence (5'-3')
4354	c.346_350dup	ACTTCCTGCTTGGGTCTCATTG	GGAGTGATCCTGGCAATGGT
5517	c.421_422delinsTA	ACTTCCTGCTTGGGTCTCATTG	GGAGTGATCCTGGCAATGGT
12701	c.441del	ACTTCCTGCTTGGGTCTCATTG	GGAGTGATCCTGGCAATGGT
13276	c.346dup	ACTTCCTGCTTGGGTCTCATTG	GGAGTGATCCTGGCAATGGT
M32	c.232C>T	CACCAGCCTAATTTGGAAAGC	CTTGACGTGATAGACGGGTTC
J0006	c.401_404dup	ACTTCCTGCTTGGGTCTCATTG	GGAGTGATCCTGGCAATGGT

Supplementary Table 3: Summary of long-read sequencing data on each subject

Sample	cell number	Total Bases	Reads Number	Mean length	Max Length	N50	Sequencing depth(X)
J0007	1	45,963,877,064	2,081,215	22,085	391,598	33,436	15.32
J0011	1	42,186,500,634	2,104,711	20,043	572,081	28,128	14.06
J0012	1	66,016,672,128	5,681,106	11,620	419,157	14,294	22.01

Supplementary Table 4：: Primers used for amplification and sequencing of the breakpoint junction sequences of J0012 family.

	Band Size	Forward primer sequence（5’-3’）	Reverse primer sequence（5’-3’）
F4/R4	812bp	TCTTGCTGCTTGTTATTGGTCT	GAAGGAGACGTCCTGCTAAA
F5/R5	515bp	ATCTAGCTGTGAATCCATCTGG	GCAACTCCAAGCATTTCAGG
f1/r1	660bp	TTCCTGGCTTTACCTAGAAG	TTGCAGTAAGTACTCTCTCA
f2/r2	570bp	AAGAAAGAGGAAGGGGAGGA	AGAGTGACACGGTGAGTGGT
